# Activation of Soluble Adenylyl Cyclase Protects against Secretagogue Stimulated Zymogen Activation in Rat Pancreaic Acinar Cells

**DOI:** 10.1371/journal.pone.0041320

**Published:** 2012-07-23

**Authors:** Thomas R. Kolodecik, Christine A. Shugrue, Edwin C. Thrower, Lonny R. Levin, Jochen Buck, Fred S. Gorelick

**Affiliations:** 1 Section of Digestive Diseases, Department of Internal Medicine, Yale University School of Medicine, New Haven, Connecticut, United States of America; 2 Department of Cell Biology, Yale University School of Medicine, New Haven, Connecticut, United States of America; 3 Veterans Administration Connecticut Healthcare, West Haven, Connecticut, United States of America; 4 Department of Pharmacology, Weill Medical College of Cornell University, New York, New York, United States of America; University of Oldenburg, Germany

## Abstract

An early feature of acute pancreatitis is activation of zymogens, such as trypsinogen, within the pancreatic acinar cell. Supraphysiologic concentrations of the hormone cholecystokinin (CCK; 100 nM), or its orthologue cerulein (CER), induce zymogen activation and elevate levels of cAMP in pancreatic acinar cells. The two classes of adenylyl cyclase, trans-membrane (tmAC) and soluble (sAC), are activated by distinct mechanisms, localize to specific subcellular domains, and can produce locally high concentrations of cAMP. We hypothesized that sAC activity might selectively modulate acinar cell zymogen activation. sAC was identified in acinar cells by PCR and immunoblot. It localized to the apical region of the cell under resting conditions and redistributed intracellularly after treatment with supraphysiologic concentrations of cerulein. In cerulein-treated cells, pre-incubation with a trans-membrane adenylyl cyclase inhibitor did not affect zymogen activation or amylase secretion. However, treatment with a sAC inhibitor (KH7), or inhibition of a downstream target of cAMP, protein kinase A (PKA), significantly enhanced secretagogue-stimulated zymogen activation and amylase secretion. Activation of sAC with bicarbonate significantly inhibited secretagogue-stimulated zymogen activation; this response was decreased by inhibition of sAC or PKA. Bicarbonate also enhanced secretagogue-stimulated cAMP accumulation; this effect was inhibited by KH7. Bicarbonate treatment reduced secretagogue-stimulated acinar cell vacuolization, an early marker of pancreatitis. These data suggest that activation of sAC in the pancreatic acinar cell has a protective effect and reduces the pathologic activation of proteases during pancreatitis.

## Introduction

The exocrine pancreas responds to a meal by secreting digestive zymogens (particularly proteases, such as trypsinogen and chymotrypsinogen) into the small intestine, where they are converted to active enzymes. However, during the early phases of acute pancreatitis, a severe inflammatory disease of the pancreas, these zymogens are prematurely activated within the pancreatic acinar cell and have a central role in promoting injury. The intracellular signaling molecule, cAMP, has been found to enhance secretagogue-sensitive zymogen activation when intracellular cAMP levels are increased using membrane permeable analogs of cAMP [1], [2]. This response was also observed after treatment with secretin, vasoactive intestinal peptide (VIP) and pituitary adenylate cyclase-activating peptide (PACAP), whose receptors are linked to plasma membrane adenylyl cyclases [2]. This enhancement of activation was mediated by both cyclic-AMP dependent protein kinase (PKA) and exchange protein directly activated by cyclic AMP (EPAC) [3]. Stimulation of acinar cells with the physiologic ligand cholecystokinin (CCK) at supraphysiologic levels (10–100x physiologic) or its orthologue cerulein (CER), increases cAMP levels and cellular PKA activity [4]. Further, Rap1, a small GTP binding protein activated by cAMP stimulation of EPAC, modulates acinar cell amylase secretion [5] and is associated with zymogen granule membranes [6], [7]. Although these studies demonstrated effects of cAMP on acinar cell responses, they did not examine the potential contribution by specific cAMP pools on pancreatitis responses.

cAMP can be produced either by transmembrane adenylyl cyclases (tmAC) or by the recently characterized soluble adenylyl cyclase (sAC) [8], [9], [10]. sAC was originally described as having two variants, a 187 kD full-length form (sACfl) and a truncated 48 kD product of alternative splicing (sACt) [11]. The full-length protein consists of 2 catalytic subunits, which are most closely related to those found in cyanobacteria and mycobacteria, a consensus P-loop, and a leucine zipper sequence. sACt consists of only the 2 catalytic subunits and is approximately 20-fold more active than the full-length form [12]. Neither sACfl nor sACt contain a membrane-spanning domain [10]. Additional variants of sAC have recently been described [13] including a somatic form of sAC that arises from an alternate start site preceding exon 5 [14]. Although sAC was originally isolated from the testis, it has been found in other tissues [15], [16] including the pancreas [15]. sAC is predominately cytosolic, but can also be associated with cellular organelles such as the nucleus, mitochondria and microtubules [17]. sAC was originally described as a bicarbonate (HCO_3_) sensor [18], but Ca^2+^ and other ions can also stimulate its activity [19], [20], [21]. A combination of HCO_3_ and Ca^2+^ activates sAC synergistically [20]. sAC has also been shown to be activated by changes in intracellular pH [22]. Changes in Ca^2+^ and intracellular pH have been shown to play pathophysiological roles during pancreatitis [23], [24], [25]. Further, multiple mechanisms, including carbonic anhydrase IX [26], Na-HCO_3_ co-transporter [27], [28], Cl-HCO_3_ exchanger [27], [28] and CFTR [29], can modulate HCO_3_ levels and therefore, sAC, in the acinar cell.

An issue related to cAMP signaling is potential degradation by cytoplasmic phosphodiesterases prior to reaching its cell targets. One mechanism that circumvents the need for cAMP diffusion is signal compartmentalization. Such restriction of cAMP production has been observed in various cell types including cardiac myocytes [30], [31], [32], COS-7 cells [33], HEK-293 cells [34]and N1E-115 neuroblastoma cells [35]. These localized cAMP signals are likely due to its generation by distinct cytosolic and membrane-associated sAC and provides a mechanism whereby cAMP effector molecules can be selectively activated.

The identification of sAC offers an alternative to simple diffusion from sites of generation: that of localized cAMP production and signaling [17], [33]. The close association of sAC with target molecules has been shown for PKA [33] and for the EPAC/Rap1 pathway [19], [21], [36]. To determine whether sAC might affect acinar cell responses, we examined its role in secretagogue-stimulated zymogen activation. sAC was identified in acinar cells and found to mediate CER-induced cAMP elevations. Further, the cellular responses to sAC-derived cAMP appeared to be in part due to PKA activation. The activation of sAC with bicarbonate was found to cause the inhibition of secretagogue-stimulated zymogen activation; this effect was partially reversed by inhibition of either sAC or PKA. Finally, we demonstrated that bicarbonate treatment reduced formation of large vacuoles, an early feature of acute pancreatitis. Our data suggest a potentially protective role for sAC in secretagogue stimulated zymogen activation in the rat pancreatic acinar cell.

## Results

### sAC is Present in Pancreatic Acinar Cells

**Figure 1:**
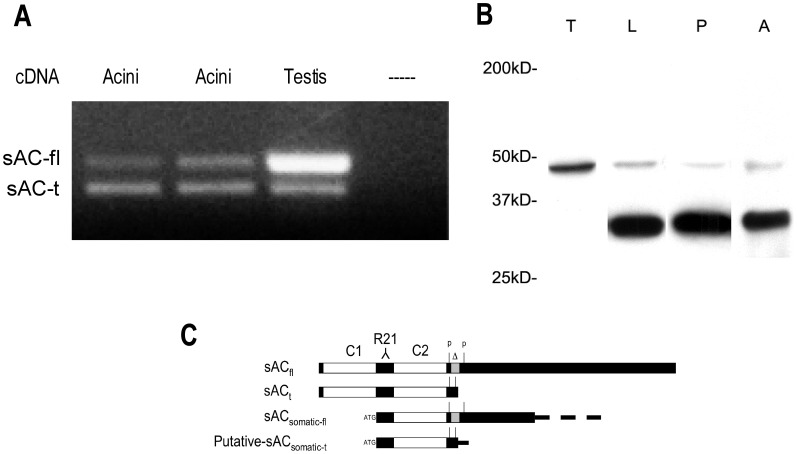
sAC is present in the pancreatic acinar cell. (A) RNA was isolated from pancreatic acini and the testis and PCR was performed using primers, which can distinguish full length (sAC-fl) from truncated sAC (sAC-t). Duplicates of cDNA from two different preparations of acini are shown (B) Immunoblot for sAC using sAC Ab (R21). Lanes: T:testis, L:liver and P:pancreas A:acini (50 µg protein/lane). (C) Diagram adapted from Wuttke et.al. [51] and Farrell et.al. [14] of sAC isoforms including full length (sACfl), truncated (sACt), somatic (sACsom) and a putative somatic truncation (putative sACsomtic-t). Also indicated are the PCR primer sites (P), the site of the 53 nt deletion(Δ), the site of Ab R21 recognition (R21) and the two catalytic domains (C1 and C2).

The presence of sAC in pancreatic acinar cells was first determined by PCR and immunoblot (Fig. 1A, 1B). Primers were used to amplify a region that contains an alternate splice site: a 56 nt deletion that results in a premature stop and the truncated form of sAC [11] as detailed in Figure 1C. Two PCR products were found in the acinar cell corresponding to full length and truncated sAC; the truncated form of sAC appeared to be more abundant than the full-length form (Fig. 1A).

Immunoblot analysis of other tissues using an antibody specific for sAC (R21) [17] has previously identified bands at 187 and 50 kD corresponding to sACfl and sACt, respectively. This antibody labeled a single band of approximately 48 kD in testis (Fig. 1B, Lane T). In rat liver, the 48 kD band as well as a dominant 34 kD band was identified (Fig. 1B, Lane L). In both whole rat pancreas (Fig. 1B, Lane P) and rat pancreatic acinar cells (Fig. 1B, lane P) we observed only 2 bands: a faintly labeled 48 kD band and a predominant 34 kD band. The full-length 187 kD form was not detected in any of the tissues; this may be due to low copy number compared to the other transcripts. The presence of a 34 kD band has been described in colonic epithelial cells [37] and the liver [38]. The 34 kD band found in both the pancreas and liver may be a truncation of a previously described somatic sAC (Fig. 1C). This is consistent with our PCR data given that both somatic and germ cell sAC contain the sequence to which our PCR primers are targeted. The locations of the PCR primers, deletion site and antibody epitope, as well as the relative configuration of the various sAC isoforms, are shown in Figure 1C. These findings provide molecular evidence that sAC is present in pancreatic acinar cells.

### sAC is Localized to Both Cytosolic and Membrane Compartments in the Pancreatic Acinar Cell and this Localization Changes with CER Stimulation

**Figure 2:**
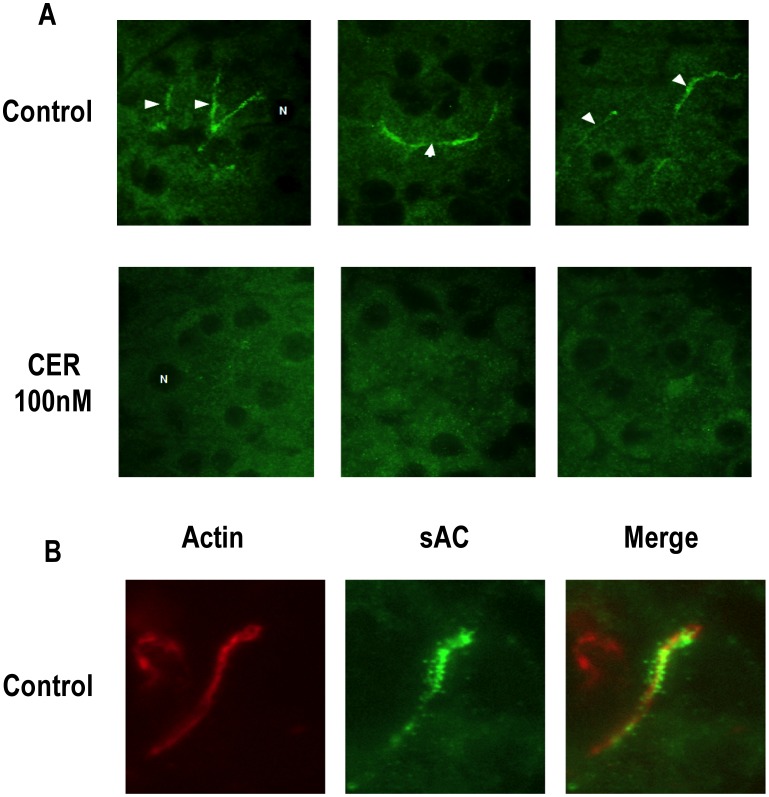
sAC is localized to the apical pole of acinar cell and undergoes translocation after cerulein (CER) stimulation. Tissue was removed from control and CER treated rats and was probed for sAC and f-actin. (A) Pancreatic tissue was probed with antibodies to sAC (R21). sAC immunoreactivity was concentrated in the apical regions of the acinar cell (arrow heads), but labeling was also observed in the cytoplasm in control tissue (CTL). In CER hyperstimulation (CER) there was a loss of apical staining and the appearance of intense cytoplasmic puncta. 3 representative pictures from different experiments are shown. Representative nuclei are marked with and “N”. B) Control tissue probed for both sAC (green) and also f-actin (rhodaminne-phalloidin, red). In the merged co-localization image of sAC with f-actin is yellow.

**Figure 3:**
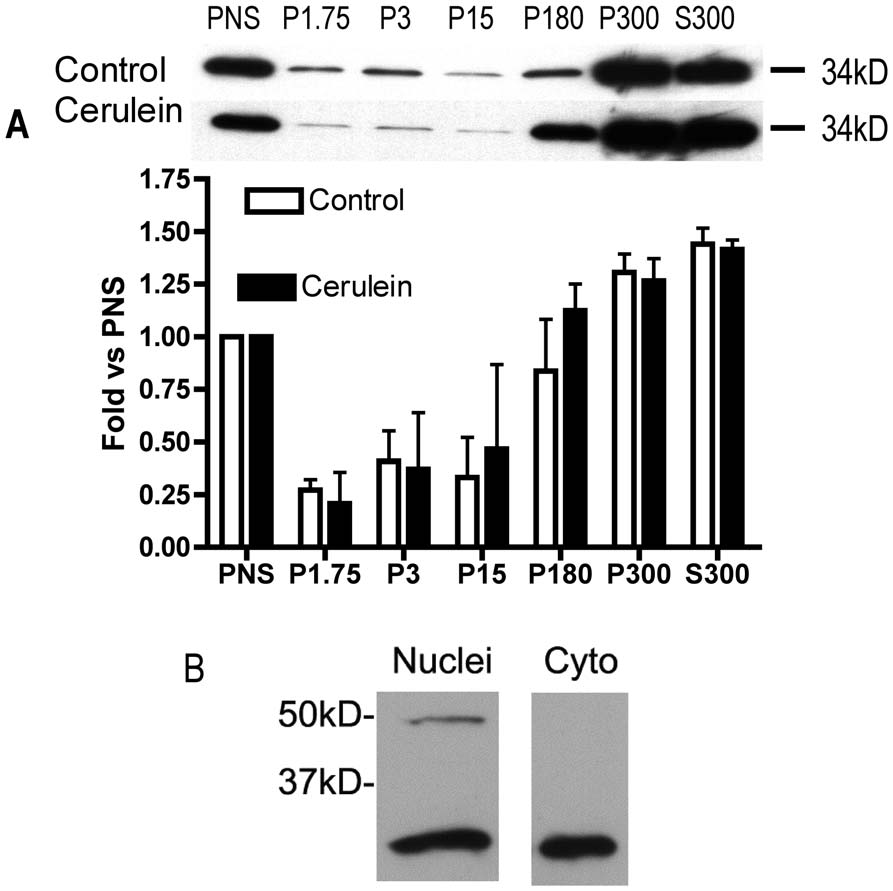
sAC is associated with specific subcellular fractions. (A) Tissue was removed from control and CER treated rats and separated by differential centrifugation into various membrane fractions or cytosol followed by Immunoblot analysis of the 34 kD band of sAC. The top panel shows representative immunoblots and below is quantitation of all experiments (n = 3). (B) Immunoblot for sAC using Ab (R21) in cytoplasmic and nuclear protein extract from pancreatic acini (n>3).

When sections from unstimulated pancreas were examined by immunofluorescence (IF) labeling using antibody R21 raised against sAC, the labeling concentrated in a compartment (2A) that was localized just below the apical actin cytoskeleton (2B). This labeling appeared to have a vesicular component (2B). Faint labeling of punctate throughout the cytoplasm was also observed (2A). After CER hyper-stimulation, there was a loss of labeling at the apical pole of the acinar cell (Fig. 2A) Fractionation of pancreas (from unstimulated rats and animals treated with CER to induce pancreatitis) by differential centrifugation showed that the majority of the sAC (34 kD) was found in the cytosol (Fig. 3A, S300) and very light membrane fractions (Fig. 3A, P180 and P300), with much less sAC associated with heavier membrane fractions (Fig. 3A, P1.75, P3 and P15). Although we observed little nuclear staining in our pancreatic sections by IF, sAC was also found by immunoblot in nuclei isolated from acini (Fig. 3B). These findings demonstrate that sAC has a distinct sub-cellular distribution in the basal state and is redistributed when the acinar cell is treated with supraphysiologic concentrations of CER.

### sAC, But not tmAC Inhibition, Enhances CER Stimulated Zymogen Activation and Amylase Secretion

**Figure 4:**
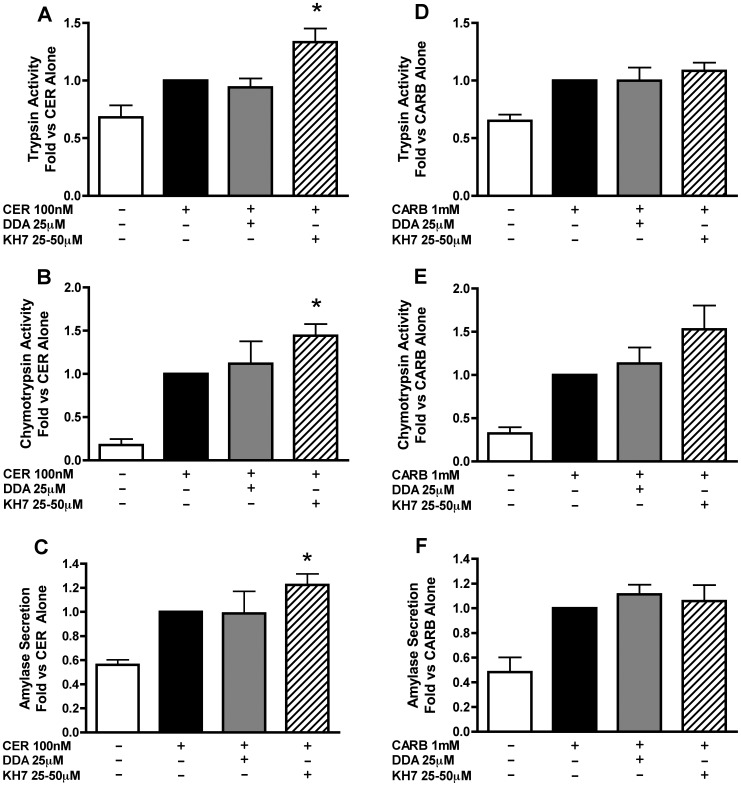
Inhibition of sAC, but not tmAC, enhances cerulein (CER)-stimulated zymogen activation and amylase secretion, but not CARB stimulated responses. Acini were treated with or without the sAC inhibitor KH7 (25–50 µM) for 2 hours or the tmAC inhibitor DDA [25 µM] for 1 hour prior to the addition of either CER [100 nM] or CARB [1 mM]. Cells were assayed after one hour of secretagogue treatment for trypsinogen activation (A,D), chymotrypsinogen activation (B,E) and amylase secretion (C,F). *p<0.05 vs. corresponding CER or CARB treatment alone (n≥3).

Previous studies have shown that stimulation of acinar cells with supraphysiological concentrations of CCK [4], [39], but not the muscarinic agonist, carbachol (CARB) [39], [40], leads to an increase in cellular cAMP levels. The differences between these two pancreatitis stimuli provide an ideal system for elucidating cAMP-specific effects in the acinar cell. To determine whether CCK-induced zymogen activation is due to activation of sAC or tmAC, we used specific inhibitors and the cholecystokinin (CCK) orthologue, CER. Due to variations in potency among sAC inhibitor KH7 preparations, each was added at a concentration that was pre-determined to cause maximal enhancement of zymogen activation [25–50 µM]; the tmAC inhibitor DDA was added at 25 µM. Neither inhibitor had any significant effect on zymogen activation alone (data not shown). Inhibition of sAC caused a significant increase in CER-stimulated activation of both trypsinogen and chymotrypsinogen (Fig. 4A–B), but tmAC inhibition had no significant effect on either zymogen (Fig. 4A–B). sAC inhibition also enhanced CER-stimulated amylase secretion (Fig. 4C).

CARB has been reported not to stimulate cAMP accumulation in pancreatic acinar cells [39]. Inhibition of tmAC with DDA had no effect on CARB-stimulated zymogen activation (Fig. 4D–E) or amylase secretion (Fig. 4F). Inhibition of sAC resulted in a slight, but insignificant, increase in CARB-stimulated chymotrypsinogen activation (Fig. 4E), but did not affect CARB-stimulated trypsinogen activation (Fig. 4D) or amylase secretion (Fig. 4F). These findings suggest that sAC is selectively activated by supraphysiologic concentrations of CER and are also consistent with CARB having little to no effect on cAMP production in acinar cells.

### Inhibition of PKA Enhances Secretagogue-stimulated Zymogen Activation and Amylase Secretion

**Figure 5:**
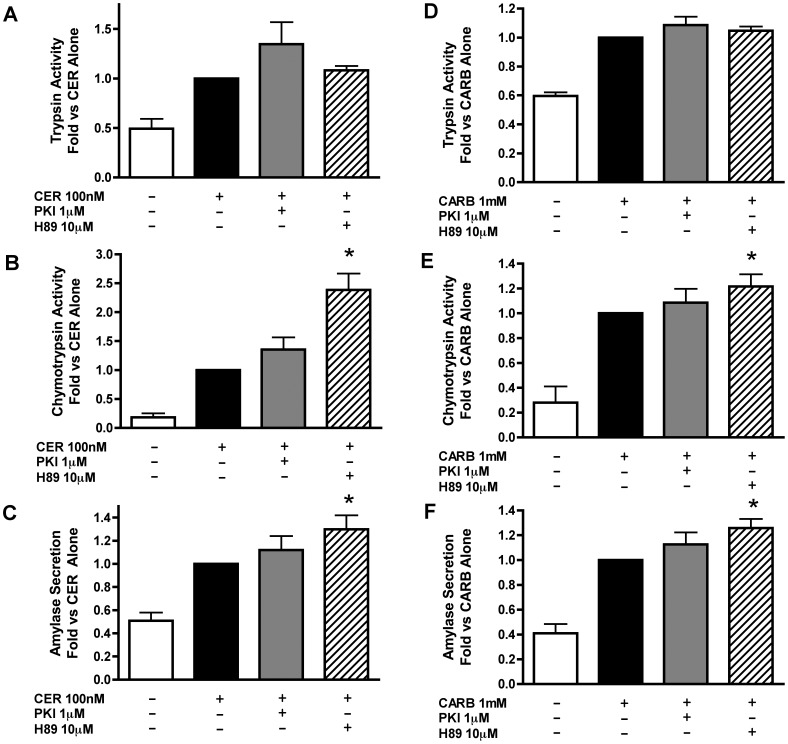
Inhibition of PKA enhances secretagogue stimulated zymogen activation and amylase secretion. Acini were treated with or without the PKA inhibitor PKI [1 µM] or H89 [10 µM] for 60 min prior to the addition of either CER [100 nM] or CARB [1 mM]. Cells were assayed after one hour of secretagogue treatment for trypsinogen activation (A,D), chymotrypsinogen activation (B,E) and amylase secretion (C,F). *p<0.05 vs. corresponding CER or CARB treatment alone (n≥5).

To determine whether the effects of secretagogue-stimulated cAMP were PKA mediated, the effects of two different PKA inhibitors, PKI and H89, were examined. Neither inhibitor alone affected zymogen activation or amylase secretion (data not shown). Similar to the results obtained with sAC inhibition, we found that PKA inhibition with PKI tended to increase secretagogue stimulated trypsinogen (Fig. 5A,D) and chymotrypsinogen (Fig. 5B,E) activation. To confirm the role of PKA using another class of kinase inhibitor, cells were pretreated with H89 (10 µM). This agent caused a significant increase in secretagogue stimulated chymotrypsin activation (Fig. 5B,E), but had no effect on trypsinogen activation (Fig. 5A,D). PKA inhibition with H89 also resulted in a small, but significant, enhancement of amylase secretion (Fig. 5C,F). Together, these findings support an inhibitory role for PKA in both secretion and zymogen activation.

### Bicarbonate Activation of sAC Inhibits Secretagogue-stimulated Zymogen Activation and Amylase Secretion and is Reversed by Inhibition of sAC but not tmAC’s

**Figure 6:**
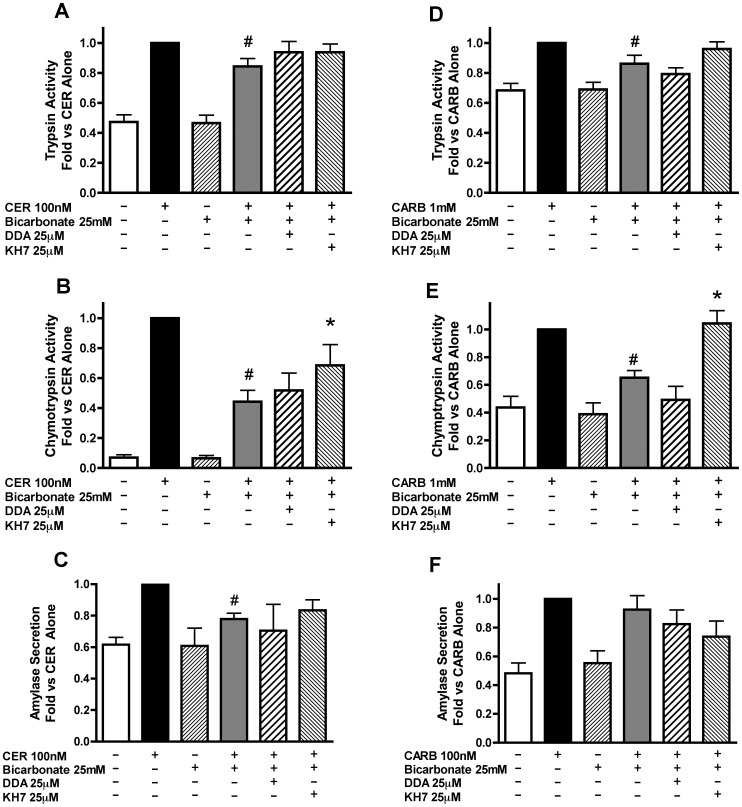
Activation of sAC with bicarbonate inhibits secretagogue stimulated zymogen activation and amylase secretion and is partially blocked by sAC inhibition but not tmAC inhibition. Acini were treated with or without either the sAC inhibitor KH7 [25–50 µM] for 2 hours or the tmAC inhibitor DDA [25 µM] for 1 hour prior to changing media and replacing it with either 25 mM bicarbonate at a constant flow of air/CO_2_ (95/5%) or without the addition of bicarbonate under room air conditions. Secretagogue (CER 100 nM or CARB 1 mM) was added immediately after media change to the appropriate wells and acini incubated for 1 hour. Acini were collected and assayed for trypsinogen activation (A,D) and chymotrypsinogen activation (B,E) and amylase secretion (C,F). #p<0.05 vs. corresponding treatment without bicarbonate *p<0.05 vs. corresponding bicarbonate/secretagogue treatment (n≥5).

To determine whether sAC activation would have the opposite effects of its chemical inhibition, the effects of stimulating the enzyme with HCO_3_ on secretagogue stimulated zymogen activation were examined. Acini were incubated in buffer with or without 25 mM sodium bicarbonate, a concentration shown to increase sAC-mediated secretion in cholangiocytes [41]. Since acinar cell responses are highly sensitive to changes in extracellular pH [42], the concentration of HEPES in all buffers was adjusted to 25 mM to maintain a constant pH. In addition, buffers containing bicarbonate were bubbled with air/CO_2_ (95%/5%) for 30 min to allow for the equilibration of the pH and gas content. Bicarbonate treatment inhibited both CER-(Fig. 6A–B) and CARB-(Fig. 6D–E) stimulated zymogen activation as well as CER-stimulated amylase secretion (Fig. 6C). The effects of bicarbonate on chymotrypsin activity were substantially greater than that observed with trypsin. We next examined whether AC inhibition would impact the bicarbonate effects using DDA to inhibit tmAC’s and KH7 to inhibit sAC. tmAC inhibition did not change the effect of bicarbonate on either secretagogue-stimulated zymogen activation (Fig. 6A,B and D,E) or amylase secretion (Fig. 6C,F). sAC inhibition did not significantly change the bicarbonate effect on secretagogue-stimulated trypsinogen activation, (Fig. 6A,D). However, sAC inhibition partially reversed the effect on CER-stimulated chymotrypsinogen activation (Fig. 6B) and almost completely reversed the effects of bicarbonate on CARB-stimulated chymotrypsinogen activation (Fig. 6E). KH7 did not have a significant effect on the inhibition of CER-stimulated amylase secretion (Fig. 6C). Although bicarbonate likely affects many ion transport mechanisms, these findings and those shown below strongly suggest that sAC is a key mediator of bicarbonate effects on zymogen activation.

### Effects of sAC Modulation on Basal and Secretagogue Stimulated cAMP Accumulation

**Figure 7:**
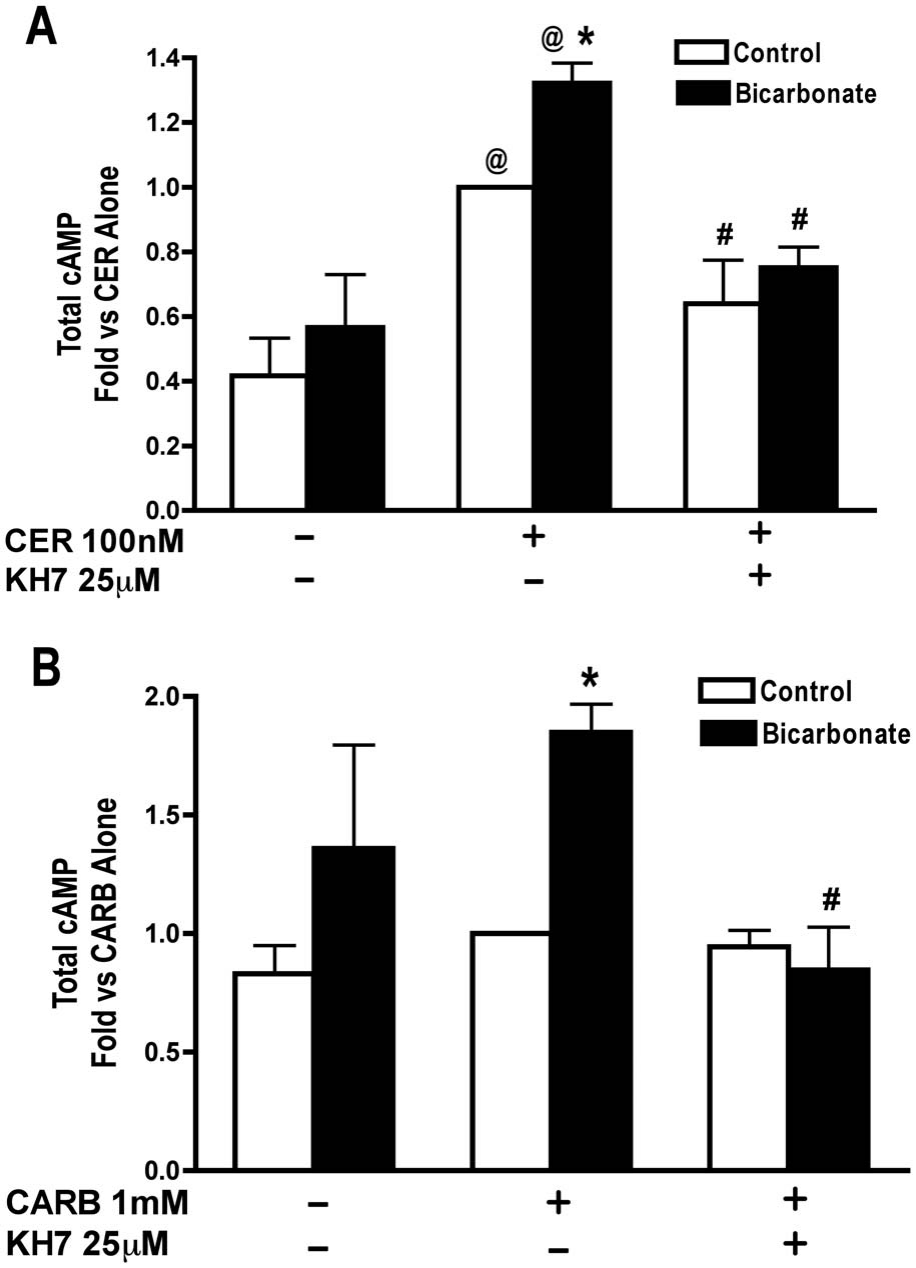
Secretagogue stimulated cAMP accumulation is enhanced by bicarbonate treatment and is inhibited by the sAC inhibitor KH7. Acini were treated with or without either the sAC inhibitor KH7 [25 µM] for 2 hours. During the last 15 min, IBMX was added [1 mM]. Media was changed and inhibitors where re-added when appropriate. Media used for the change contained either bicarbonate [25 mM] at a constant flow of air/CO_2_ (95/5%) or no added bicarbonate under room air conditions. Secretagogue, CER 100 nM (A) or CARB 1 mM (B) was added immediately after media change to the appropriate wells and acini incubated for 1 hour. Acini were collected and assayed for cAMP accumulation. *p<0.05 comparing treatments with and without bicarbonate, #p<0.05 vs corresponding treatment without KH7, @ p<0.05 vs corresponding control without secretagogue. (n≥4).

We next examined the effects of bicarbonate and sAC inhibition on secretagogue stimulated cAMP accumulation. The ability of CER to cause cAMP accumulation was confirmed (Fig. 7A) and CARB caused a slight, but non-significant increase in cAMP (Fig. 7B). Bicarbonate treatment significantly enhanced cAMP accumulation when combined with either CER (Fig. 7A) or CARB (Fig. 7B) stimulation. Pretreatment with the sAC inhibitor, KH7, inhibited CER-stimulated cAMP accumulation (Fig. 7A), and also inhibited the increase in cAMP accumulation due to bicarbonate treatment in CER (Fig. 7A) and CARB (Fig. 7B) treated cells. The sAC inhibitor KH7 may have effects on cAMP levels in control (1.00 vs 1.40+/−0.26, p = 0.17: fold vs control) and bicarbonate (1.664+/−0.81 vs 0.51+/−0.17, p = 0.26: fold vs control) treated acini, but these differences were not statistically significant. Together, these data suggest that CER-stimulated as well as bicarbonate-enhanced, cAMP accumulation is due to activation of sAC.

### PKA Inhibition Partially Reverses the Effect of Bicarbonate on Secretagogue Stimulated Zymogen Activation and Amylase Secretion

**Figure 8:**
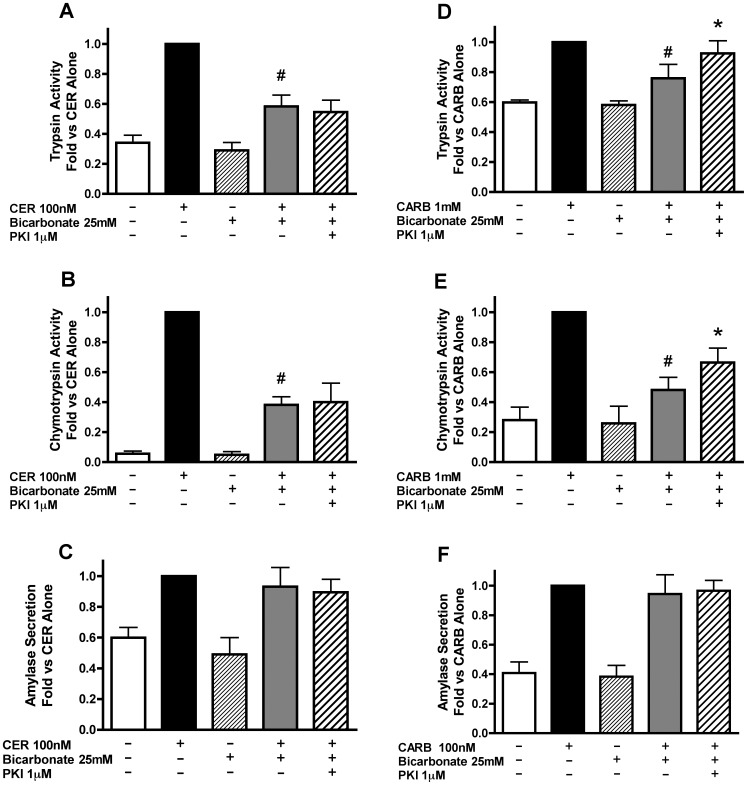
The effect of bicarbonate on secretagogue stimulated zymogen activation decreased by PKA inhibition using myristolated PKI. Acini were treated with or without the PKA inhibitor PKI [1 µM] for 60 min prior to changing media and replacing it with either 25 mM bicarbonate and a constant flow of air/CO_2_ (95/5%) or no added bicarbonate under room air conditions. Secretagogue (CER 100 nM or CARB 1 mM) was added immediately after media change to the appropriate wells and acini incubated for 1 hour. Acini were collected and assayed for trypsinogen activation (A, D), chymotrypsinogen activation (B,E) and amylase secretion (C,F). #p<0.05 vs. corresponding secretagogue alone, *p<0.05 vs. corresponding secretagogue/bicarbonate treatment (n≥5).

**Figure 9:**
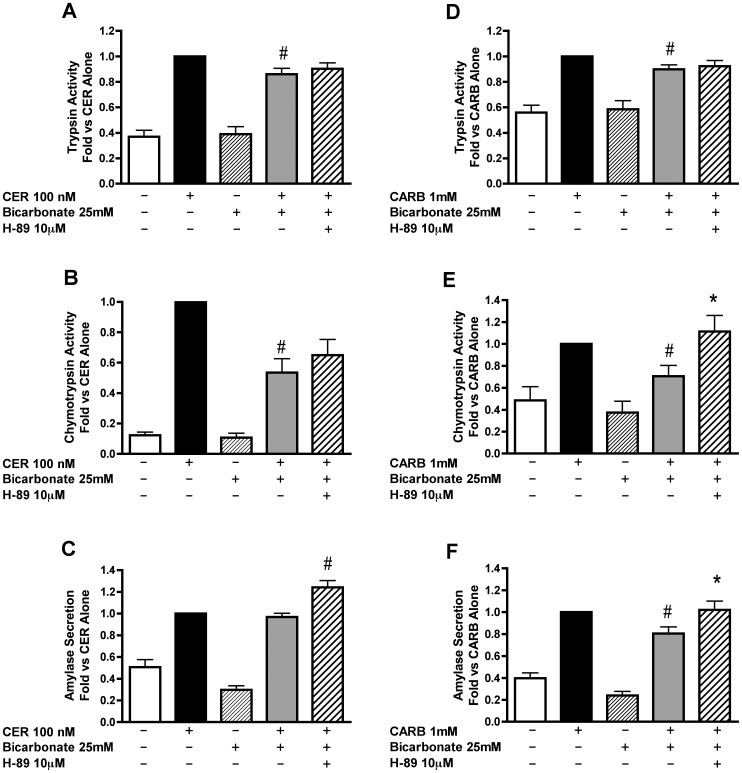
The effect of bicarbonate on secretagogue stimulated zymogen activation is decreased by PKA inhibition using H89. Acini were treated with or without the PKA inhibitor H89 [10 µM] for 30 min prior to changing media and replacing it with either bicarbonate [25 mM] at a constant flow of air/CO_2_ (95/5%) or without the addition of bicarbonate under room air conditions. H89 was added back to the appropriate wells. Secretagogue (CER 100 nM or CARB 1 mM) was added immediately after media change to the appropriate wells and acini incubated for 1 hour. Acini were collected and assayed for trypsinogen activation (A,D), chymotrypsinogen activation (B,E) and amylase secretion (C,F). #p<0.05 vs. corresponding secretagogue alone, *p<0.05 vs. corresponding secretagogue/bicarbonate treatment (n≥5).

Pharmacological inhibition was used to determine whether the increase in cAMP levels due to bicarbonate treatment mediates the inhibition of zymogen activation and secretion through PKA. PKA inhibitors, PKI or H89, had no significant effect on bicarbonate-induced inhibition of CER-stimulated zymogen activation (Fig. 8A–B, Fig. 9A–B). Although there was no effect of PKI on CER-stimulated amylase secretion (Fig. 8C), H89 enhanced CER-stimulated amylase secretion in the presence of bicarbonate (Fig. 9C) in a manner similar to the effects of H89 on CER alone (Fig. 5C). These data suggest that PKA activation does not play a role in bicarbonate inhibition of CER-stimulated zymogen activation, but modulates amylase secretion.

In CARB treated cells, PKA inhibition with PKI partially reversed bicarbonate-induced inhibition of both trypsinogen (Fig. 8D) and chymotrypsinogen (Fig. 8E) activation. However, unlike PKI, H89 had no effect on bicarbonate-induced inhibition of CARB-stimulated trypsinogen activation (Fig. 9D). H89 completely reversed the effect of bicarbonate on CARB-stimulated chymotrypsinogen activation (Fig. 9E) similar to that seen for the sAC inhibitor KH7 (Fig. 6D,E). The differential effect on these zymogens seen in figures 5, 6 and 9 may be due to the two enzymes having different activation profiles. Following CER treatment, trypsinogen activation starts earlier (15 min) than chymotrypsinogen activation (30 min) and, although chymotrypsinogen activation continues to increase with time, trypsinogen activation reaches a maximum (30 min) and then falls [43]. Additional findings suggest that trypsinogen and chymotrypsinogen might be activated by distinct mechanisms as suggested by their different requirements for ATP [44]. Bicarbonate inhibited CARB-stimulated amylase secretion and this effect was reversed with H89 (Fig. 9F). These data indicate that PKA activation modulates the inhibition of CARB-stimulated zymogen activation and secretion induced by bicarbonate, but this cAMP signal likely affects other targets, such as EPAC.

### Bicarbonate Differentially Affects Markers of Cellular Injury During Secretagogue-Stimulated Zymogen Activation

**Figure 10:**
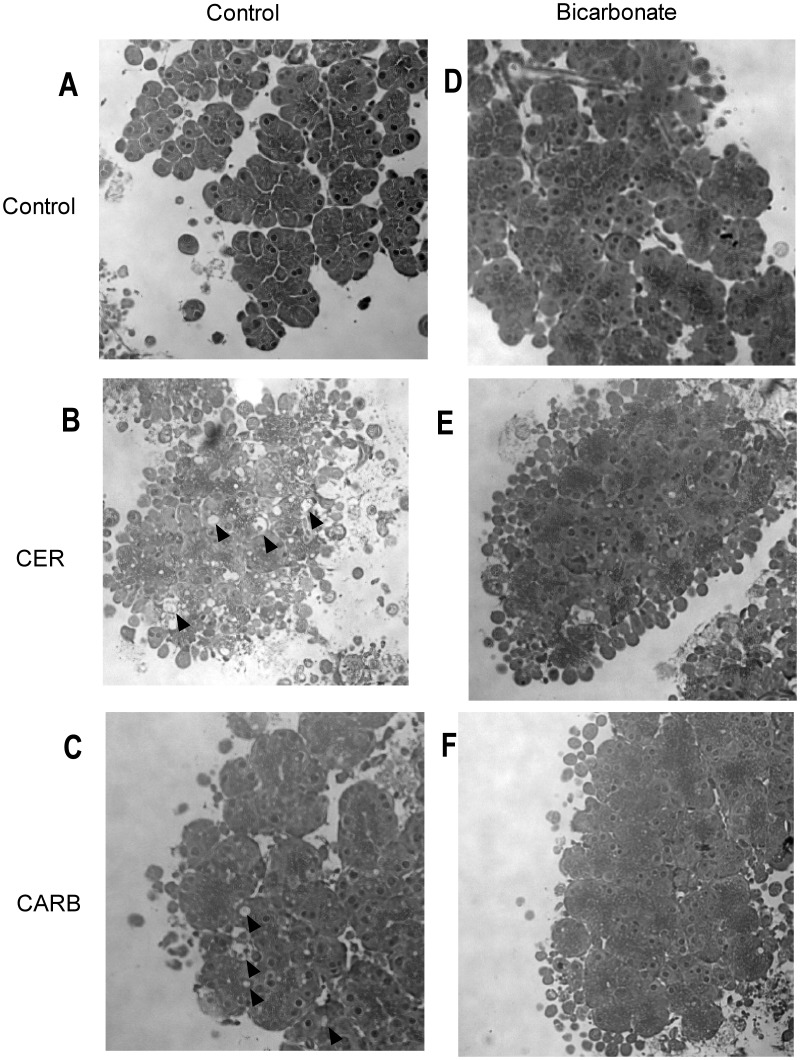
Bicarbonate treatment reduces acinar cell vacuole formation but not blebbing. Acini were treated with or without bicarbonate (25 mM) either alone or in the presence of either CER 100 nM or CARB 1 mM for 1 hour. Cells were collected and fixed in PLP fixative followed by embedding in EPON. Sections were cut and stained with hematoxylin and examined microscopically. Control (A), CER (B), CARB (C), Bicarbonate (D), CER+Bicarbonate (E) and CARB+Bicarbonate (F). Each is a representative photograph (40x magnification). Vacuoles are indicated by arrowheads.

**Figure 11:**
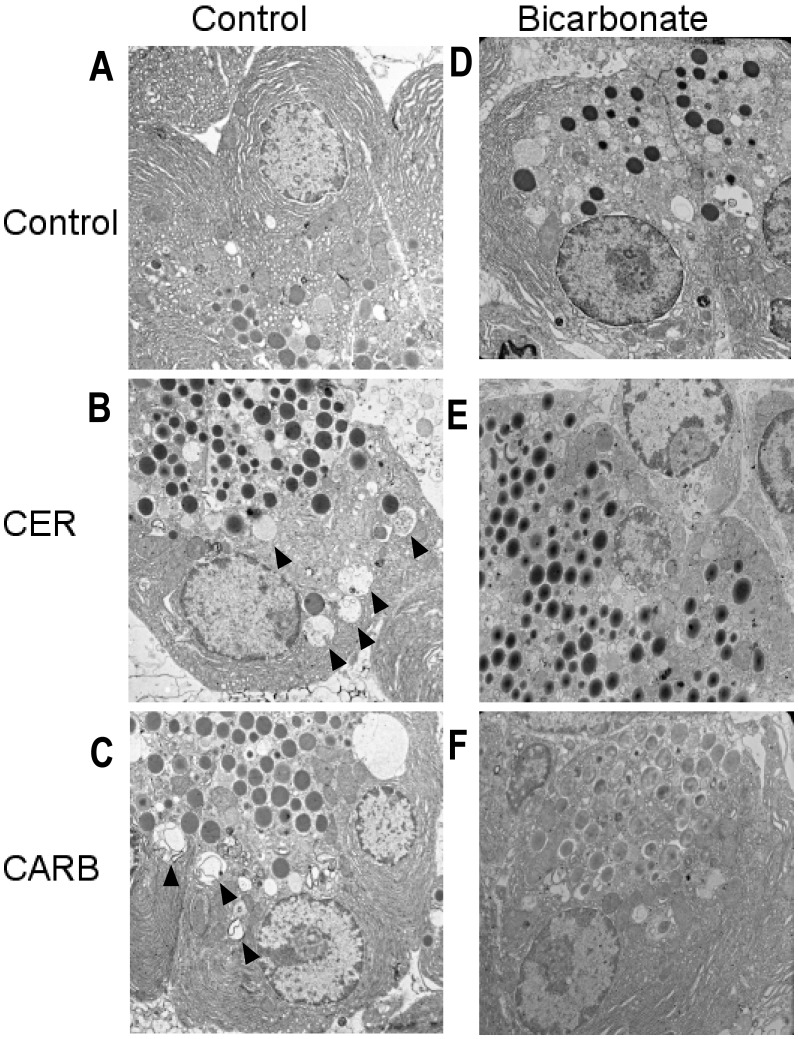
Bicarbonate treatment reduces acinar cell cytosolic vacuoles induced by hyperstimulation. Acini were treated with or without bicarbonate 25 mM either alone or in the presence of either CER 100 nM or CARB 1 mM for 1 hour. Cells were collected and fixed in PLP fixative followed by embedding in EPON. Sections were cut and examined via EM. Control (A), CER (B), CARB (C), Bicarbonate (D), CER+Bicarbonate (E) and CARB+Bicarbonate (F). Each is a representative photograph. Vacuoles are indicated by arrowheads.

**Figure 12:**
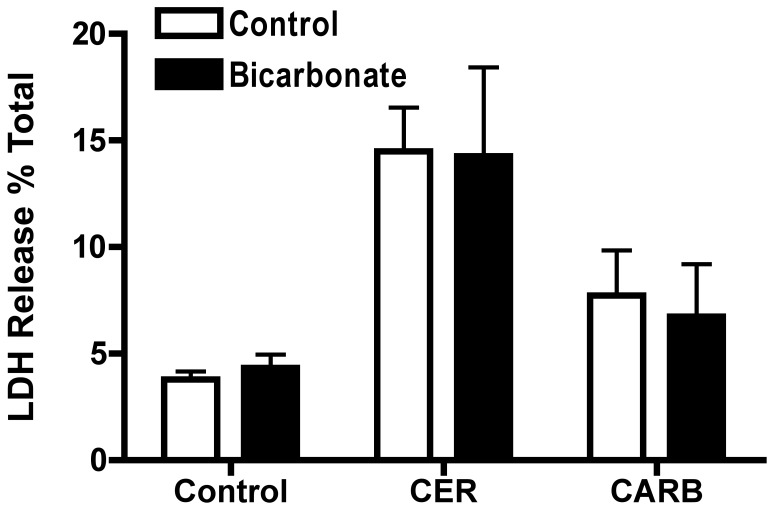
Bicarbonate treatment has no effect on secretagogue stimulated LDH release. Acini were treated with or without bicarbonate (25 mM) either alone or in the presence of either CER 100 nM or CARB 1 mM for 2 hours. Cells were collected and a cell free fraction obtained. Both the cell-free and cell-containing fractions were assayed for LDH and the results expressed as the percentage of LDH released into the media. (n≥3).

Zymogen activation is often linked to cell injury. To determine whether bicarbonate treatment would impact cell injury, we examined its effects on markers of cellular damage including morphology (membrane blebbing and vacuole formation) and LDH release. Bicarbonate treatment prominently decreased the number of large cytoplasmic vacuoles in both CER-(Fig. 10E, Fig. 11E) and CARB-(Fig. 10F, Fig. 11F) treated cells compared to CER (Fig. 10B, Fig. 11B) and CARB (Fig. 10C, Fig. 11C) alone. Both CER (Fig. 10B) and CARB (Fig. 10C) caused an increase in membrane blebbing compared to control (Fig. 10A) and bicarbonate appeared to slightly increase membrane bleb formation under all conditions (Fig. 10D–F). Finally, LDH release increased with both CER and CARB treatment and this injury response was not affected by the addition of bicarbonate (Fig. 12). These findings suggest that bicarbonate-induced activation of sAC may reduce the formation of large cytoplasmic vacuoles, a key morphologic feature of acute pancreatitis. Since these structures could be the site of zymogen activation, these findings may relate the effects of bicarbonate on protease activity.

## Discussion

The current study demonstrates that sAC is present in the exocrine pancreas and suggests that it can modulate the activation of digestive enzymes in the pancreatic acinar cell, a key initiating event in acute pancreatitis. These studies also establish a precedent by demonstrating that cAMP generated by membrane AC can elicit different responses in an epithelial cell than those seen with sAC. Since the effect of activating sAC in the acinar cell is protective with respect to zymogen activation, it could represent an attractive therapeutic target.

We find that pancreatic acinar cells have both full-length and truncated forms of sAC as detected by PCR (Fig. 1A). By immunoblot, we identified two immunoreactive bands, including a 48 kD and 34 kD species (Fig. 1B) that have also been described in mouse liver [38] and guinea pig colonic epithelial cells [37]. Based on the PCR results and the site of the antigen recognized by the R21 antibody used for immunoblot (Fig. 1C), we predict that the prominent 34 kD band may be a truncation of the previously described somatic soluble adenylyl cyclase (Fig. 1C). It has been shown that isolated catalytic domains of tmAC (C1 and C2), exhibit no cyclase activity and cannot be activated individually. However, when these domains are reconstituted *in vitro*, low-level cyclase activity is detected. Furthermore, upon treatment with forskolin and/or Gs-alpha, cAMP levels are produced by the reconstituted C1/C2 complex, similar to those seen with the native form [45]. We hypothesize that the mechanism by which the truncated form of somatic sAC produces cAMP may be similar, whereby the truncated somatic sAC, containing only C2 associates with a cytosolic C1 domain thereby forming an active complex. The association of C1 with C2 as distinct modules would also suggest a potential form of regulation in the acinar cell.

Using immunofluorescence we observed a change in sAC localization with CER stimulation from an apical and cytosolic distribution to a punctuate intracellular pattern (2A), Cell fractionation studies showed sAC to be associated with various membrane fractions, but these studies did not confirm the CER stimulated redistribution seen by immunofluorescence. This discrepancy may arise due to 1) the discontinuous nature of differential centrifugation (i.e. the inability to discern small changes in density); 2) movement of apical membrane structures to an intracellular location; 3) translocation of sAC between compartments of similar density. Furthermore, sAC was also detected in isolated nuclei by immunoblot in unstimulated acinar cells. In the nuclei of HeLa cells and rat liver, sAC colocalizes with both the regulatory subunit of PKA and phospho-CREB [33]. Though not a topic of the present study, it is possible that a nuclear pool of sAC in the acinar cell could modulate transcriptional responses.

The addition of cell permeable analogs of cAMP or the stimulation of AC-linked receptors has been shown to enhance secretagogue stimulated zymogen activation and amylase secretion [1], [2], [3] in acinar cells. These treatments likely resulted in the activation of cAMP targets that differ in localization and activity from those activated by sAC. CCK at hyperstimulatory concentrations [∼0.1 µM] causes an increase in total cAMP levels in acinar cells [4], but local concentrations of cAMP could be much higher because its diffusion is limited by phosphodiesterase-dependent degradation. Since sAC can be activated by calcium, a potential mechanism for sAC activation in acinar cells is by secretagogue-stimulated elevations in intracellular calcium. We examined this possibility using calcium free media in combination with chelation of intracellular calcium with BAPTA, but found no effect on CER stimulated cAMP accumulation (data not shown). This suggests that activation of sAC in the acinar cell is not due to increases in intracellular calcium, but occurs by another undetermined mechanism.

Inhibition of sAC results in an increase in CER-stimulated zymogen activation. This was surprising since previous results showed cAMP enhanced CER-stimulated zymogen activation [2]. Our data also suggests that PKA may be downstream of sAC activation. This response may reflect the production of highly localized levels of cAMP that may otherwise be overlooked when assaying total cellular cAMP levels. Together, our findings suggest that activation of tmAC enhances zymogen activation, whereas activation of sAC in the acinar cell inhibits zymogen activation.

We found that activation of sAC with bicarbonate decreased CER-stimulated zymogen activation. Inhibition of PKA, downstream of sAC, blocked bicarbonate effects, but only on CARB-stimulated zymogen activation, with no effect seen for CER. One possible explanation is that the elevated cAMP levels caused by CER stimulation could also act through EPAC as well as PKA. This is consistent with published data suggesting that cAMP derived from sAC activation leads to the activation of Rap1 via EPAC [19], [21], [36]. Activation of EPAC and its target Rap1 has also been shown to be involved in amylase secretion from pancreatic acinar cells [5]. Alternately, another non-PKA or EPAC related pathway such as a cyclic nucleotide gated channel, similar to that gated by cGMP, might be involved [46].

Given the protective effect of bicarbonate treatment on secretagogue stimulated zymogen activation, we examined its effect on secretagogue stimulated cellular injury. When cells were treated with CER or CARB, typical early morphologic features of acute pancreatitis, plasma membrane blebbing and cytosolic vacuolization were observed indicating cellular damage. One marker of cell injury, LDH release, was unaffected by treatment with bicarbonate. Previous studies showed, that a change in secretagogue stimulated zymogen activation did not always result in a change in LDH release [1]. The addition of bicarbonate did not reduce membrane blebbing, but appeared to decrease the formation of large cytoplasmic vacuoles. Many of these vacuoles appeared to be autophagic in etiology and contained partially digested membranes and organelles. In other studies, autophagy has been functionally linked to zymogen activation [47], [48], [49]. Whether sAC and bicarbonate are affecting zymogen activation through effects on autophagy will be the focus of future studies.

The findings presented here could have important clinical implications. Resting serum levels of bicarbonate, a sAC activator, are 25 mM in humans and can fall well below 15 mM in patients with pancreatitis. Further, the levels of serum bicarbonate correspond to the severity of pancreatitis: the lower the value, the more severe the disease [50]. Since the kM of sAC for bicarbonate is ∼15 mM, it is likely that sAC would be active in the basal state, but may fall below this threshold during pancreatitis. Since a mainstay treatment for those at risk for developing acute pancreatitis, or those presenting with the disease, has been fluid resuscitation, the levels of bicarbonate in these intravenous fluids could have clinical relevance. Resuscitation fluids vary widely in their bicarbonate content. Our data suggest a potential beneficial role for bicarbonate-containing buffers in the treatment of patients presenting with acute pancreatitis or the prophylactic use of bicarbonate-rich solutions in those at high risk for developing this disease.

In conclusion, this study shows that sAC is present in the acinar cell and that cAMP derived from sAC activation has a distinct and unexpected role in regulating secretagogue-mediated pancreatitis responses, the most prominent being zymogen activation.

## Materials and Methods

### Ethics Statement

All experiments and procedures using animals (100 rats total) were approved by the Veterans Administration Institutional Animal Care and Use Committee (IACUC), West Haven, CT (Veterans Administration Public Health and Safety, Office of Laboratory Animal Welfare Assurance # A4363-01).

### Preparation of Isolated Pancreatic Acini

Pancreatic acini were isolated as described [2]. Briefly, fasted male Sprague-Dawley rats 100–125 g (Charles River Laboratories, Wilmington, MA) were euthanized by CO_2_. The pancreas was minced in buffer-A: 10 mM Hepes (pH 7.4), 95 mM NaCl, 4.7 mM KCl, 0.6 mM MgCl_2_, 1 mM NaH_2_PO_4_, 10 mM glucose, 2 mM glutamine, plus 0.1% bovine serum albumin, 1× MEM-amino acids (GIBCO-BRL, San Jose, CA)] and washed 2 times. Cells were then digested for 1 hour at 37°C in buffer-A containing 50 U/ml of type-4 collagenase (Worthington, Freehold, NJ) under O_2_ with constant shaking. The digest was filtered through a 300–400 µm mesh (Sefar American, Depew, NY) and the resulting groups of acinar cells, acini, were distributed in a 24-well Falcon tissue culture plate (Becton Dickinson, Franklin Lakes, NJ). All reagents were purchased from Sigma Biochemical (St. Louis, MO) unless otherwise noted.

### Models of Secretagogue Pancreatitis in Isolated Pancreatic Acinar Cells

In this study, we used groups of pancreatic acinar cells, also called acini, treated with either cerulein (CER) or carbachol (CARB) to elicit pancreatitis responses. Under physiologic stimulation (CER 0.1 nM and CARB 1 µM) these ligands stimulate maximal amylase secretion, but little to no intracellular zymogen activation. Acini were stimulated with supraphysiologic concentrations (100–1000 fold high than physiologic; CER 100 nM and CARB 1 mM) of ligands to induce pancreatitis responses.

### Incubation of Acinar Cells with Bicarbonate

To maintain a constant pH in a bicarbonate/CO_2_ system for these experiments, buffer-A was supplemented with HEPES to a final concentration of 25 mM (Buffer B). Cells were isolated as above and recovered for 2 hours with a media change at 1 hour. After recovery, the media was changed with either buffer-B or buffer-B supplemented with 25 mM sodium bicarbonate (Mallinckrodt Baker inc, Phillipsburg, NJ), which had been equilibrated with air/CO_2_ (95%/5%) for 30 min and pH adjusted to 7.4. In some treatment groups, AC or PKA inhibitors were added during the recovery period as described below. Inhibitors were re-added after the media change at the end of the recovery period. Cells in media supplemented with bicarbonate were incubated under air/CO_2_ (95%/5%) and cells without bicarbonate were incubated under room air during secretagogue treatment. Secretagogue (CER 100 nM or CARB 1 mM) was added for 1 hour. After 1 hour of treatment, the contents of the wells were transferred to 1.5 ml Eppendorf tubes and centrifuged 30× g for 1 min. After centrifugation, 50 µl of cell free media was removed to assay for amylase secretion. The tubes containing the cell pellets and remaining media as well as those containing media alone were stored at −80°C until used to assay zymogen activation and amylase secretion.

### Treatment of Acinar Cells with Adenylyl Cyclase (AC) Inhibitors

Cells were isolated as above. Cells were recovered for 2 hours and during this recovery period cells were pretreated with AC inhibitors. Cells were treated with either the sAC inhibitor KH7 [25–50 µM] for 2 hours or the tmAC selective inhibitor 2′, 5′-Dideoxyadenosine (DDA; 25 µM) (Calbiochem a division of EMD Chemicals Inc, Gibbstown, NJ) for 1 hour. After recovery the media was exchanged for new media with inhibitors. Secretagogue (CER 100 nM or CARB 1 mM) was then added for 1 hour. After 1 hour of treatment the contents of the wells were transferred to 1.5 ml Eppendorf tubes and treated as described above.

### Treatment of Acinar Cells with Protein Kinase A (PKA) Inhibitors

After isolation, cells were recovered for 2 hours with a media change at one hour. After the media change, either the PKA inhibitor PKI (1 µM) (PKA Inhibitor 14–22 Amide, Cell-Permeable, Myristoylated, Calbiochem a division of EMD Chemicals Inc, Gibbstown, NJ) was added for the last hour of recovery or H89 [10 µM] (Sigma, St. Louis, MO), a dose previously used in pancreatic acini [5] to inhibit PKA, was added for the last 30 min of recovery. Following recovery, the media was exchanged and PKA inhibitors were re-added at the same concentration. Secretagogue (CER 100 nM or CARB 1 mM) was then added for 1 hour. After 1 hour of treatment, the contents of the wells were processed for zymogen activation and amylase secretion assays as described previously.

### Protease Activity Assays in Acinar Cells

Samples frozen at −80°C were thawed in ice, and homogenized in 1.5 ml centrifuge tubes with a pestle and centrifuged at 1,000 *g* for 1 min. The resulting post nuclear supernatant was assayed for protease activity using fluorogenic substrates as described [1]. Briefly, 50 µl of enzyme substrate (40 mM final) (chymotrypsin; Calbiochem a division of EMD Chemicals Inc, Gibbstown, NJ and trypsin; Peptides International, Louisville, KY) was added to each well containing 100 µl of sample and 350 µl of assay buffer (50 mM Tris (pH 8.1), 150 mM NaCl, 1 mM CaCl_2_) and read using a fluorometric plate reader (HTS 7000; Perkin-Elmer Analytical Instruments, Shelton, CT or Flx800, BioTek instruments, Winooski, VT) at excitation wavelength 380 nm and emission 440 nm for 20 measurements over 10 min. The slope of the resulting line, which represents enzyme activity of the homogenate, was normalized to amylase content.

### Amylase Assay

Samples were thawed on ice and the cell-free supernatant was assayed for secreted amylase. The remaining sample (cells plus medium) from the zymogen activation assays was assayed for total amylase. Amylase activity was determined by using a commercial kit (Phaebadas kit; Magle Life Sciences, Lund, Sweden). Amylase secretion was calculated as the percent total release (medium/medium+cells).

### cAMP Assay

Cells were incubated as above for bicarbonate treatment with the following changes. The phosphodiesterase inhibitor IBMX was added (1 mM) for the last 15 min of recovery and was re-added after the last media change. After treatments, cells were collected and 5 µl of concentrated HCl was added to block phosphodiesterase activity and cAMP levels were assayed in the extract using a cAMP Direct EIA kit from Assay Designs (Ann Arbor, MI) according to the manufacturer’s directions.

### Polymerase Chain Reaction (PCR)

Total RNA was isolated from rat pancreatic acini and testis using the RNeasy Midi kit (Qiagen, Valencia, CA). First strand cDNA was prepared with oligo(dT) as the primer using the Superscript II kit (Invitrogen, Carlsbad, CA) following the manufacturer’s protocol. PCR was carried out using 1 µl first-strand cDNA in a 50 µl reaction volume containing 20 mM Tris pH 8.4, 50 mM KCl, 2 mM MgCl_2_, 200 mM of each deoxynucleotide triphosphate, 1.25 units *Taq* DNA polymerase (Invitrogen, Carlsbad, CA) and 250 nM each of primers rsAC-3f (5′-CACGAGTACACAGTCATTGG-3′) and rsAC-3r (5′-TATGCGGCCGCTGACTTTCTCATTGAGG-3′) described previously [11]. Amplification conditions were an initial denaturation for 3 min at 94°C then 40 cycles of denaturation (94°, 45 sec), annealing (57°C, 30 sec), and extension (72°C, 30 sec). This was followed by a single extension step for 10 min at 72°C. PCR products were analyzed on agarose gels that contained ethidium bromide.

### Subcellular Fractionation

Tissue was harvested from control animals and animals treated *in vivo* with CER (40 µg/kg body weight) for 1 hour prior to tissue collection. Tissue was first crudely chopped and then gently homogenized in Homogenization Buffer 1(HB1; 0.3 M sucrose, 10 mM Tris pH 6.6) using a Potter-Elvehjem grinder at low speed. If subcellular fractions were to be used for immunoblots, protease inhibitors were added to HB1: Complete Protease Inhibitor Mini, EDTA-free cocktail (1 tablet per 15 ml stock solution; Roche, Mannheim, Germany), 5 mM benzamidine, 0.1 mg/mL soybean trypsin inhibitor. Pancreas homogenate was centrifuged at 500 *g* for 10 min at 4°C and the pellet re-suspended in additional HB1 and re-centrifuged. The resulting supernatants were pooled (PNS) and centrifuged at sequential spins (1750 *g*, 3000 *g*, 15,000 *g*, each 10 min, 4°C and 180,000 *g*, 300,000 *g*, each 1 hr, 4°C) onto a 2 M sucrose cushion. The supernatant from the 300,000 *g* spin (cytosol) and the pellets from each spin were assayed for protein concentration for immunoblot.

### Immunoblot

Tissues were harvested from rats and processed for subcellular fractionation as described above or were homogenized in 0.3 M sucrose, 10 mM HEPES (pH 7.4), 5 mM benzamidine and 1X complete protease inhibitor cocktail (Roche Diagnostics, Indianapolis, IA). Homogenates were centrifuged 500 g for 15 min to generate a post-nuclear supernatant (PNS). Nuclear and cytoplasmic extracts were isolated from acinar cells using a commercially available kit (NE-PER kit, Thermo Scientific, Rockford, IL). Protein concentration was determined using a Pierce 660 nm protein assay (Pierce, Rockford, IL). Samples (50 µg protein/lane) were separated on SDS-PAGE gels (AnykD, Bio-Rad, Hercules, CA) and transferred to Immobilon-P membranes (Millipore, Billerica, MA). Membranes were blocked for 1 h at room temperature with Blotto (TBS, 5% nonfat dry milk, 0.05% Tween-20). Membranes were then probed with primary antibody (1∶1500) in Blotto for 1 h at room temperature, washed and then probed with horseradish peroxidase-labeled goat anti-mouse IgG (Sigma, St. Louis, MO) for 1 hour at room temperature. After treatment with the secondary antibody, membranes were washed in TBS, and autoradiography was performed by using a SuperSignal West Pico chemiluminescence kit (Pierce, Rockford, IL).

### Immunofluoresence

Tissue was harvested from control animals and animals treated *in vivo* with CER (40 µg/kg body weight) and cut into small (∼2–3 mm) cubes, and frozen in OTC compound (Sakura Finetek, Torrance, CA). Frozen sections were cut fixed on the slide with 4% paraformaldehyde in phosphate buffered saline (PBS). Sections were then labeled with a monoclonal Ab raised against sAC (R21) or double labeled for sAC and f-actin (phalloidin). In brief, samples were permeabilized with PBS containing 0.1% triton X-100 (IF buffer) for 15 min followed by quenching in IF buffer with 0.5 M ammonium chloride for 10 min. After quenching, slides were washed 3 times with IF buffer and then blocked for 1 hour with IF buffer with 2% BSA (blocking buffer). After blocking, slides were denatured for 4 min in IF buffer +1% SDS. Slides were washed 3 times with IF buffer and primary Ab (R21) was added (1∶500) in blocking buffer over night at 4°C. The next day slides were washed 3 times with IF buffer Slides were incubated with 0.5 M NaCl for 10 min followed by 3 washes. To those slides stained for f-actin, 100 nM rhodamine-phalloidin (Cytoskeleton Inc, Denver, CO) was added for 30 min followed by 3 washes of IF buffer. Secondary Ab was then added (goat anti-mouse IgG labeled with Alexa 488; Molecular Probes, Eugene, OR) for 1 hour. Slides were again washed 3 times with IF buffer and then mounted using Vectashield hard set mounting media with DAPI (Vector Laboratories, Burlingame, CA). Images were captured using a Spot camera mounted on a Zeiss axiophote fluorescent microscope.

### Lactic Dehyrogenase (LDH) Assay

LDH assays were performed using a Cytotox 96 nonradioactive cytotoxicity assay kit according to the manufacturer’s instructions (Promega, Madison, WI). In brief, acinar cells were treated as above, with or without bicarbonate (25 mM), either alone or in the presence of CER (100 nM) or CARB (1 mM) for 2 hours. Samples were collected and centrifuged at 30 *g* for 1 min. A 50 µl aliquot of medium was then removed to measure LDH release from the cells. A manufacturer-provided lysis reagent was then added to the remaining 450 µl of cells and medium to determine total LDH. Both cell and medium samples were assayed. The results are expressed as the percent LDH released into the medium.

### Cell Morphology

Isolated cells were treated with or without bicarbonate (25 mM), alone or in the presence of either cerulein (CER) 100 nM or carbachol (CARB) 1 mM for an hour. Cells were pelleted 30 g for 5 min and media was removed. To the cell pellets, 1 ml of PLP fixative (10 mM NaIO_4_, 75 mM Lysine, 37.5 mM NaPO_4_, 2% paraformaldehyde) was added and placed on ice for 1 hour. Pellets were then washed 2 times with PBS and post-fixed in 1.0% osmium tetroxide (Polysciences, Inc, Warrington, PA), dehydrated in ethanol in propylene oxide, embedded in 100% of the epoxy resin EPON, and sectioned using an ultramicrotome. Sections stained with hematoxylin/eosin and examined for plasma membrane blebbing microscopically using a Zeiss axiophote microscope. Cytosolic vacuolization was assessed using transmission electron microscopy (EM).

### Statistical Analysis

Data represent the mean ± SE of at least 3 individual experiments unless otherwise noted, with each performed in at least duplicate. Students *t*-test analysis was used to determine statistical significance and p values <0.05 were assigned significance.
